# Correction: Magnetic control of cellular processes using biofunctional nanoparticles

**DOI:** 10.1039/c7sc90067h

**Published:** 2017-11-01

**Authors:** Cornelia Monzel, Chiara Vicario, Jacob Piehler, Mathieu Coppey, Maxime Dahan

**Affiliations:** a Institut Curie , PSL Research University , Laboratoire Physico Chimie , CNRS UMR168 , UPMC , F-75005 Paris , France . Email: maxime.dahan@curie.fr; b University of Osnabrück , Department of Biology/Chemistry , Division of Biophysics , 49076 Osnabrück , Germany

## Abstract

Correction for ‘Magnetic control of cellular processes using biofunctional nanoparticles’ by Cornelia Monzel *et al.*, *Chem. Sci.*, 2017, 8, 7330–7338.



## 

The authors regret that there was an error in ref. 74 of the original article. The correct reference, in which the article number has been corrected, is presented herein as ref. 1.

The Royal Society of Chemistry apologises for these errors and any consequent inconvenience to authors and readers.
